# Increasing Vaccination: Psychological Characteristics of COVID-19 Vaccine Advocates, Converts, and Resisters in Hong Kong

**DOI:** 10.3390/vaccines10101744

**Published:** 2022-10-19

**Authors:** Xiaohui Wang, Yi-Hui Christine Huang, Qinxian Cai

**Affiliations:** Department of Media and Communication, City University of Hong Kong, Hong Kong 999077, China

**Keywords:** COVID-19, vaccination, psychological factors, trust, longitudinal survey

## Abstract

This study uses longitudinal data to profile psychological characteristics of COVID-19 vaccine advocates, resisters, and converts. We conducted a two-wave longitudinal survey (*N*wave1 = 3190, *N*wave2 = 2193) in Hong Kong using stratified quota sampling. Among those who completed both survey waves, 458 (30.5%) were classified as vaccine advocates, 295 (19.7%) were vaccine resisters, and 621 (41.4%) were vaccine converts (who shifted away from hesitancy). Compared to advocates, resisters were more likely to be female, those without children, between 40 and 49 years old, democratic voters, and those with poor health. Highly educated individuals, non-democrats, and those in good health were more likely to convert from hesitancy to acceptance. Public trust in authorities and confidence in vaccine were the primary factors related to vaccine uptake. Those who were more confident in vaccine, those who increased in information consumption and risk perceptions towards the pandemic, and those who decreased in their trust of health professionals were more likely to convert. Our study complements the emerging global picture of COVID-19 vaccine acceptance by focusing on changes in vaccine hesitancy during the pandemic.

## 1. Introduction

The COVID-19 pandemic is expected to disrupt daily life until an effective vaccine is widely accepted. There is growing global concern about vaccine hesitancy—the widespread reluctance to take safe and recommended vaccines [1,2]. Vaccine hesitancy becomes more complex as new SARS-CoV-2 variants emerge and new vaccines come to market. Several studies have documented COVID-19 vaccine hesitancy and acceptance using cross-sectional data [2,3,4,5,6,7]. Less is known, however, about how vaccine hesitancy changes to acceptance.

Promoting vaccine uptake requires an understanding of those who are willing to be vaccinated, why they are willing to do so, and their decision-making process. Vaccine hesitancy was already a growing concern before the COVID-19 pandemic [8]. A 5C framework developed from previous research describes five main psychological barriers for vaccination behavior [8,9,10], including confidence (safety and effectiveness of vaccines, acceptability of vaccine-related risks), complacency (not perceiving diseases as high risk), risk calculation (engagement in extensive information gathering), convenience (structural or psychological barriers to conversion), and collective responsibility (the willingness to protect others via one’s own vaccination).

Another model for increasing vaccinations offers three general focus areas for increasing vaccine uptake based on a systematic review of psychological factors related to vaccination [11]. The first is individuals’ thoughts and feelings, referring to risk perceptions of infectious disease, confidence in the effectiveness and safety of vaccines, and trust in public health systems and officials. The second is social processes, referring to the influence of interpersonal or more complex population-level social interactions. The third focus area is practical conditions, such as convenience, cost, and quality of service. 

One key driver of vaccine acceptance appears to be concern about vaccine safety and effectiveness, reflecting the rapid pace of vaccine development and worries about mild, yet common and transient side effects [2,12]. This is related to another major determinant: trust in government, health authorities, and healthcare workers [12,13]. Arguably, trust of the source of information is an intrinsic and potentially modifiable component of successful uptake of vaccines [14,15]. The COVID-19 pandemic has been characterized by intense politicization and conspiracy theories [16], which may have intensified feelings of distrust shown towards information from government and health authorities [17,18,19].

An understanding of the determinants of vaccine hesitancy is necessary in order to shift it towards vaccine acceptance. This study provides novel insights into psychological factors that modulate vaccination decisions. Building on the 5C framework, we propose a modified framework that includes the following elements as determinants of vaccine uptake: individuals’ vaccine confidence, pandemic risk perception, information consumption, trust in government and health officials, and sociodemographic factors. We moved beyond documenting vaccine hesitancy rates to collect longitudinal data that captured changes in a population’s intentions to vaccinate. To our knowledge, this is the first study to track individuals’ changes towards the COVID-19 vaccine during the pandemic. We analyzed three groups of individuals—vaccine advocates, resisters, and converts—to help authorities customize effective communication. A key implication of this study is the importance of increasing acceptability

## 2. Materials and Methods

### 2.1. Data Collection

**Figure 1:**
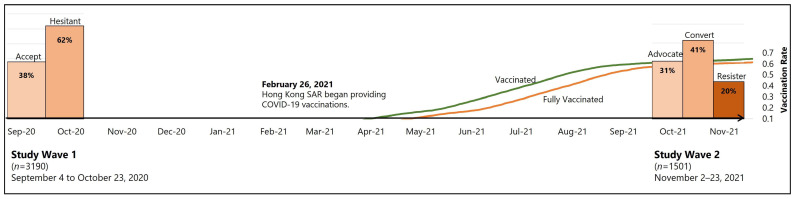
First wave data were collected through stratified sampling based on age, gender, and residential districts from 4 September to 23 October 2020, when COVID-19 vaccines were not available in Hong Kong. Second wave data were collected from 2–23 November 2021, when vaccination rates in Hong Kong plateaued and third-dose vaccines became available. These two waves of data fully capture psychological changes before and after vaccine availability. **Vaccine advocates** are defined as those who demonstrated intent to vaccinate in wave 1 and reported being fully vaccinated in wave 2. **Vaccine converts** are those who were hesitant to vaccinate in wave 1 but reported being fully vaccinated in wave 2. **Vaccine resisters** are those who were hesitant to vaccinate in wave 1 and reported being unvaccinated in wave 2.

Respondents were recruited by Rakuten Insight (https://member.insight.rakuten.com.hk/, accessed on 10 January 2021), an online survey agency, which manages more than 50,000 panelists in Hong Kong. Two survey waves were conducted with a 14-month interval. The first wave was conducted between September and October 2020 (N = 3190), when COVID-19 vaccines were still unavailable in Hong Kong. The second wave was conducted in November 2021 (N = 1501), when vaccination rates in Hong Kong plateaued. Both waves together helped us capture Hong Kong citizens’ perceptual changes before and after vaccines became available (Figure 1). In the first wave, stratified quota sampling drew on the city’s 2016 population census, based on the distribution of gender, age, and residence. The second wave was conducted in November 2021. All wave 1 participants were invited, and 68.75% (*n* = 2193) chose to participate again. Data cleaning in both waves was conducted using rigorous principles in order to ensure data quality. Specifically, respondents who failed the attention check question did not complete the two-wave survey. Similarly, we eliminated respondents who finished the questionnaire in less than one-third of the median completion time. We measured respondent demographics in both waves. The consistency of respondents’ answers for gender, age, and education was also used as a criterion for data inclusion. Specifically, if answers for gender, age, and education level from the same respondent ID deviated across the two waves, all responses from that ID were regarded as invalid. Data analysis was conducted using the final complete sample of 1501 respondents.

### 2.2. Pre-Test

The survey questionnaire consisted of items measuring individuals’ vaccine uptakes (9 items), psychological factors (30 items), and sociodemographic factors (7 items). We developed a Chinese version of the questionnaire with items adopted from previous studies [10,11,12,14,20,21,22] (see Supplementary Figure S2). The surveys were delivered in traditional Chinese, which is one of the official languages used in Hong Kong. Translations were performed and validated through the standard back-translation procedure [23], i.e., the Chinese version was back-translated into English to see whether the translation was closely related to the source items. A pre-test was conducted to ensure the survey instruments were readable, understandable, and comprehensive to yield valid responses. A total of 15 Hong Kong citizens who are experts in online survey studies were invited via the researchers’ network to complete the pre-test. Pre-test participants were first asked to comment on the survey including the relevance of the survey questions, the sequence of the questions, the survey length, etc. Issues of survey design were resolved through multiple discussions among the project’s researchers. Reliability checks for all variables in the pre-test were then conducted and the results were satisfactory. In view of those methods, the Institutional Review Board at the sponsoring university approved the study.

### 2.3. Measurements

This study examined psychological determinants of vaccine uptake. After a literature review, we incorporated individual psychological factors. These psychological factors included vaccine confidence, perceived risk of COVID-19, trust in the public health system, information consumption, and issue politicization. Definition and operationalization of these variables are detailed below (also see Supplementary Table S4). 

*Categorization of Vaccine Advocates, Vaccine Converts, Vaccine Resisters, and Others.* The categorization of the four groups was based on the participants’ responses of their vaccination intention in wave 1 when the vaccine was not available in Hong Kong and vaccination status in wave 2 when the vaccination program started. In wave 1, people’s vaccine intention was measured by asking respondents the degree to which they agreed on a 7-point scale (1 = strongly disagree and 7 = strongly agree): (1) I would get the vaccine sometime soon; (2) If I were faced with the decision of whether to get the vaccine today, I would choose to get it; (3) I would get the vaccine in the future [24]. We aggregated the responses to these three questions and divided them into three groups, i.e., willing to get vaccinated (with scores higher than 4), neutral (with scores equal to 4), and unwilling to get vaccinated (with scores lower than 4). In wave 2, people’s actual vaccination status was measured by asking participants to choose one answer from the following options: (1) I don’t plan to receive the vaccine; (2) I have scheduled to be vaccinated but have not yet been vaccinated; (3) I have had my first shot of the vaccine; (4) I have had my first dose of the vaccine (only one-dose required, e.g., J&J vaccine); (5) I’ve had my first dose of the vaccine and don’t plan to get the second dose; (6) I have had my second dose of the vaccine. 

Based on self-reported vaccination intention in wave 1 and vaccination status in wave 2, we divided participants into four groups: vaccine advocates (*n* = 458), referring to those who demonstrated intent to vaccinate in wave 1 and reported being fully vaccinated in wave 2; vaccine converts (*n* = 621), referring to those who were hesitant to vaccinate in wave 1 but reported being fully vaccinated in wave 2; vaccine resisters (*n* = 295), those who were hesitant to vaccinate in wave 1 and reported being unvaccinated in wave 2; others (*n* = 127), those who converted from intention to vaccinate in wave 1 to unvaccinated in wave 2 or those who received only one dose of vaccine.

*Vaccine Confidence.* Vaccine confidence refers to public believe in the importance, safety, and effectiveness of vaccines, along with people’s willingness to live with risks (De Figueiredo et al., 2021). This willingness arises when perceived benefit outweighs potential risk [25]. Respondents were asked how much they agree with the following three statements on a 7-point scale (1 = strongly disagree and 7 = strongly agree): (1) I can accept the potential risks of the vaccine, (2) I am highly willing to accept the potential risks of the vaccine, and (3) after careful deliberation, I think the vaccine does more good than harm (Wave 1: Mean (M) = 4.06, Standard Deviation (SD) = 1.25, Cronbach’s alpha (α) = 0.88; Wave 2: M = 4.29, SD = 1.38, α = 0.89) [20].

*COVID-19 Risk Perception*. Slovic and Peters proposed the dual-process model, which contends that people not only think about risk but feel risk as well [26]. Following suit, we differentiated risk perception into two categories, i.e., perceived risk and worry. Perceived risk represents the analytic process, which is slow, deliberate, sequential, and consciously controlled with high cognitive effort [27,28]. Worry denotes the heuristic process, which is intuitive, parallel, and automatic [26]. Items for both dimensions were measured on a 7-point scale where 1 = strongly disagree and 7 = strongly agree. Four items were adapted for measuring perceived risk, including “the coronavirus is almost ubiquitous, and the pathogenicity is high,” “there is a high probability of getting infected,” “the mortality rate of COVID-19 is high,” and “I have no confidence I can avoid the negative impacts of COVID-19” (Wave 1: M = 4.41, SD = 1.00, α = 0.80; Wave 2: M = 4.31, SD = 1.05, α = 0.83) [28]. For worry, respondents were asked to rate their level of agreement with the following statements with respect to COVID-19-related risk: (1) I feel fearful, (2) I feel sad, (3) I feel helpless, and (4) I feel worried (Wave 1: M = 4.35, SD = 1.21, α = 0.91; Wave 2: M = 4.34, SD = 1.20, α = 0.90) [28].

*Trust in State, Media, and Public Health Professionals*. We defined trust as the willingness to rely on another party to behave in a certain way in the context of COVID-19 [29]. The degree of trust in different parties/institutions was measured on a 7-point scale ranging from 1 = strongly distrust to 7 = strongly trust. We differentiated trustees into three groups, i.e., state governmental organizations, media, and public health professionals. For trust in state institutions, respondents were asked how much they trust the following: (1) state government, (2) the Food and Health Bureau, and (3) the Center for Health Protection (Wave 1: M = 3.67, SD = 1.47, α = 0.88; Wave 2: M = 3.81, SD = 1.49, α = 0.91). In addition, trust in media was assessed by asking respondents the degree to which they trust the Hong Kong media (Wave 1: M = 4.10, SD = 1.13; Wave 2: M = 3.95, SD = 1.14). Trust in health professionals was assessed by asking respondents the degree to which they trust Hong Kong public health professionals (Wave 1: M = 4.61, SD = 1.16; Wave 2: M = 4.52, SD = 1.21)

*Information Consumption*. Information consumption refers to routine use of multiple media outlets for the purpose of seeking information [30]. Information acquisition as a form of active information consumption was measured by asking participants the frequency on average per day of their accessing vaccine-related information from (1) television, (2) newspapers, (3) websites, (4) social media (e.g., WhatsApp, Facebook), and (5) short video platforms (e.g., Tik Tok) [30]. Responses were measured on a 7-point scale, with 1 = none, 4 = 2–3 h, and 7 = 5 h or more (Wave 1: M = 2.74, SD = 0.79, α = 0.62; Wave 2: M = 2.68, SD = 0.81, α = 0.66). Media attention as a form of passive information consumption was assessed by asking participants to rate how often they pay attention to information about COVID-19 when consuming (1) television, (2) newspapers, (3) websites (e.g., Yahoo), (4) social media (e.g., WhatsApp, Facebook), and (5) short video platforms (e.g., Tik Tok) [21,31]. A 7-point scale was adopted, with 1 = never and 7 = always (Wave 1: M = 3.67, SD = 1.04, α = 0.74; Wave 2: M = 3.49, SD = 0.98, α = 0.66).

*Issue Politicization*. The degree to which people perceive COVID-19 as a political issue was measured by four items on a 7-point scale including: “it is too mixed up in politics,” “due to political interests, policy decisions have favored some groups,” “it is politically motivated,” and “political considerations affect the nature of the information that the public receives about this issue” (Wave 1: M = 4.93, SD = 1.37, α = 0.93; Wave 2: M = 4.89, SD = 1.21, α = 0.92) [32,33].

### 2.4. Data Analysis

All statistical analysis was conducted using RStudio. A *p* value < 0.05 was considered significant. We performed multinominal logistic regression to examine the effect of sociodemographic factors and psychological factors on three groups with varying attitudes towards the vaccine. We performed analysis of covariances (ANCOVAs) to compare the mean difference between three groups for each psychological factor, with age, gender, income, and education as covariates. Turkey post hoc analyses were conducted for group comparisons

## 3. Results

We contracted with an online survey company to conduct a two-wave longitudinal survey of a representative sample of adults in Hong Kong. Of 1501 participants who completed the two-wave online survey, 1040 (69.3%) reported that they had been fully vaccinated, which is close to Hong Kong’s full vaccination rate (68.5%). Table 1 includes descriptive statistics for the sociodemographic factors of the sample. Age, gender, income, and education were used as demographic control variables in subsequent analyses.

### 3.1. Sociodemographic Profiles of Vaccine Advocates, Resisters, and Converts

Vaccine advocates—compared to vaccine resisters—were less likely to be female [Adjusted Odds Ratio (AOR) = 0.59, 95% Confidence Interval (CI) = 0.43, 0.81], aged between 40 and 49 (AOR = 0.54, 95% CI = 0.30, 0.97), without children (AOR = 0.57, 95% CI = 0.40, 0.81), pro-democrat (AOR = 0.46, 95% CI = 0.32, 0.66), poor (AOR = 0.51, 95% CI = 0.31, 0.86), or to consider their health condition as moderate (AOR = 0.59, 95% CI = 0.43, 0.83). At the same time, vaccine advocates were more likely to hold a bachelor degree (AOR = 1.77, 95% CI = 1.17, 2.68), have household income levels between 40 k and 60 k (AOR = 2.35, 95% CI = 1.35, 4.08 at 40 k) and above 60 k (AOR = 1.83, 95% CI = 1.03, 3.26), and to hold pro-Beijing sentiments (AOR = 4.66, 95% CI = 2.32, 9.35). 

Converts in wave 2—compared to vaccine resisters—were less likely to be between 30 and 49 years old (AOR = 0.58, 95% CI = 0.35, 0.95 for age between 30 and 39; AOR = 0.51, 95% CI = 0.30, 0.85 for age between 40 and 49), pro-democrat (AOR = 0.67, 95% CI = 0.48, 0.91), or to have poor health (AOR = 0.55, 95% CI = 0.34, 0.88). At the same time, converts were more likely to hold bachelors (AOR = 2.03, 95% CI = 1.38, 2.96) or post-graduate degrees (AOR = 1.92, 95% CI = 1.15, 3.20), and to express pro-Beijing sentiments (AOR = 2.60, 95% CI = 1.28, 5.27).

Four conditions distinguished vaccine converts from vaccine advocates: converts were more likely to be female (AOR = 1.74, 95% CI = 1.35, 2.26) and without children (AOR = 1.64, 95% CI = 1.24, 2.18), but less likely to be over 50 (AOR = 0.63, 95% CI = 0.40, 0.99) or pro-Beijing (AOR = 0.52, 95% CI = 0.35, 0.78) (see Table 2).

### 3.2. Psychological Factors Underpinning Vaccine Acceptance

**Figure 2:**
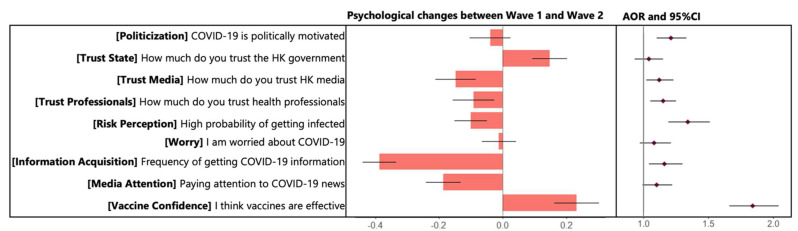
**Measured changes in psychological perceptions between two waves and association with vaccination acceptance.** Chart demonstrates Adjusted Odds Ratio (AOR) with age, gender, education, income, and health status as covariates and 95% Confidence Intervals (CIs). Hong Kong citizens showed increased trust and satisfaction with the efforts of government authorities to fight COVID-19, decreased trust in HK media, increased knowledge about COVID-19 and the vaccine, increased coping with vaccine and virus-related risks, and decreased information acquisition.

Figure 2 depicts the psychological changes observed over the course of the two-wave longitudinal survey. Generally, participants reported increased trust (M = 0.15, SD = 1.07) of state authorities, decreased trust of media (M = −0.15, SD = 1.25) and health professionals (M = −0.09, SD = 1.29), decreased risk perception (M = −0.10, SD = 1.00) and worry (M = −0.08, SD = 1.33) about COVID-19, decreased information acquisition (M = −0.39, SD = 1.05) and attention towards vaccine-related stories in the media (M = −0.19, SD = 1.06), and increased vaccine confidence (M = −0.23, SD = 1.51).

Zero-order correlations between these perceptual and attitudinal variables and vaccine acceptance, as well as the adjusted odds ratio with sociodemographic variables controlled, were examined (Supplementary Table S2). There were several non-trivial correlations between vaccination and perceptual/attitudinal factors at the zero-order and after partialing for demographics. Specifically, those who were fully vaccinated were more likely to demonstrate weaker issue politicization (AOR = 0.77), greater trust in state authorities (AOR = 1.40), stronger confidence in the vaccine (AOR = 1.33) and greater intention to vaccinate (AOR = 1.40) in wave 1. Full vaccination status was also associated with increased perceived risk of COVID-19 (AOR = 1.29) and confidence in vaccine (AOR = 1.60) across both waves. We also tested the Spearman partial correlation between psychological factors and vaccine risk acceptance. This highlighted several non-trivial correlations between issue politicization (r = −0.20), trust in state officials (r = 0.35), and information acquisition (r = 0.21) in wave 1 and vaccine confidence in wave 2. Incremental issue politicization (r = 0.13), trust in state officials (r = 0.11), trust in public health professionals (r = 0.18), and perceived risk of COVID-19 (r = 0.10) between two waves were also associated with vaccine confidence in wave 2.

### 3.3. Psychological Factors Underpinning Vaccine Acceptance

**Figure 3:**
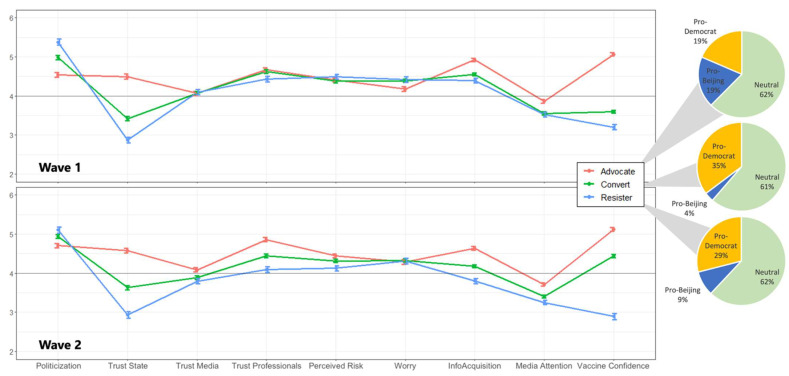
**Unadjusted means and bootstrapped standard error for all variables used in the MLR for the three vaccine profiles.** All variables were measured in a 7-point Likert scale with 1 = strongly disagree, 4 = neutral, and 7 = strongly agree. The pie charts show the distribution of political orientation within each profile. Compared to other groups, Vaccine advocates comprised more pro-Beijing (19%) individuals, while vaccine resisters were more pro-democracy (35%).

Table 3 and Figure 3 demonstrate these three vaccine profiles, which consisted of respondents with different psychological perceptions that changed over time. These differences became clearer in wave 2. In the second survey, vaccine advocates expressed relatively weak levels of issue politicization related to COVID-19, higher levels of trust in state authorities, media, and health professionals, a higher consumption of media content related to the vaccine, and higher levels of confidence in vaccine. In contrast, vaccine resisters demonstrated stronger issue politicization, lower levels of trust in state, media and health professionals, lower levels of information consumption, and lower levels of vaccine confidence. Vaccine converts fell between the advocate group and the resistance group in terms of their demonstrated issue politicization, trust levels, risk perception, rates of information acquisition, and vaccine confidence.

**Figure 4:**
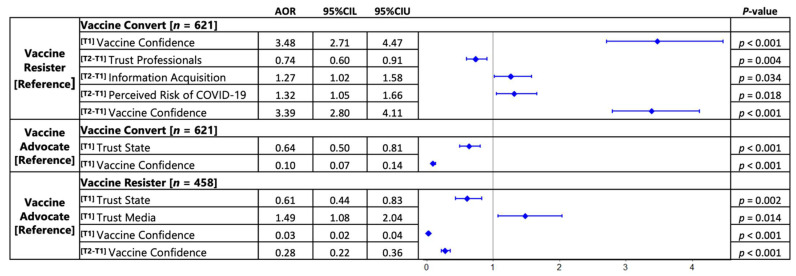
**Forest plots of results from MLR models.** AORs were adjusted for covariates of Wave 1 age, gender, education, income, number of children, and region. Factors include Wave 1 trust, information consumption, and risk perception, marked as [T1]; and their difference from Wave 2, marked as [T2-T1]. The vaccine resistant group served as a reference in both comparisons. Wald Confidence Intervals (CIs) are presented for the MLRs. Exact *p* values are presented when CIs do not include 1.

Multinomial logistic regressions showed that vaccine advocates and resisters differed mainly in their demonstrated levels of institutional trust and vaccine confidence. Compared to advocates, vaccine resisters reported significantly lower level of trust in the state (AOR = 0.61, 95% CIs = 0.44, 0.83) and higher level of trust in media (AOR = 1.49, 95% CIs = 1.08, 2.04), with lower level of confidence in vaccine (AOR = 0.03, 95% CIs = 0.02, 0.04), and had smaller increases in risk acceptance (AOR = 0.28, 95% CIs = 0.22, 0.36). Compared to advocates, vaccine converts reported significantly lower levels of trust in state authorities (AOR = 0.64, 95% CIs = 0.50, 0.81) and confidence in vaccine (AOR = 0.10, 95% CIs = 0.07, 0.14). Additionally, vaccine converts were distinguished from their vaccine resistant counterparts by being more confident in vaccine at wave 1 (AOR = 3.48, 95% CIs = 2.71, 4.47), and demonstrating decreased trust in health professionals (AOR = 0.74, 95% CIs = 0.60, 0.91), increased vaccine-related information consumption (AOR = 1.27, 95% CIs = 1.02, 1.58), increased COVID-19 risk perception (AOR = 1.32, 95% CIs = 1.05, 1.66), and an increased vaccine confidence (AOR = 3.29, 95% CIs = 2.73, 3.98) between two waves (Figure 4).

## 4. Discussion

To strengthen vaccine acceptance and reduce vaccine hesitancy, public officials should develop a better understanding of the psychological determinants of vaccination intention. Vaccine hesitancy in Hong Kong has decreased from 62.8% to 30.7% since the start of the pandemic. The pandemic also led to changes at the individual level in terms of trust in authorities, information consumption, and risk perception of both COVID-19 and the vaccine. How did such changes increase citizen’s COVID-19 vaccination uptake? This study profiles the psychology underpinning different groups of test subjects and their differing attitudes toward the COVID-19 vaccine: vaccine advocates, converts, and resisters. We offer novel evidence regarding the importance of one’s psychological beliefs in determining acceptance of the COVID-19 vaccine, with an emphasis on individuals’ confidence in vaccine and trust in authorities. Previous studies investigating the psychological characteristics related to vaccine acceptance were based on cross-sectional surveys. Our longitudinal survey measured individuals’ psychological changes over time, offering insights into why individuals convert to vaccine acceptance or continue to insist on vaccine resistance. Our results thus provide practical guidance for how to preserve current advocates and convert those who remain hesitant.

### 4.1. The Sociodemographic Profile of Vaccine Advocates, Resisters, and Converts

Results from this study indicated that, on average, vaccine advocates, vaccine resisters, and vaccine converts differed from each other in age, gender, education, income, political leaning, and self-reported health status. Males were more likely to be vaccine advocates than resisters, a finding sharply at odds with a number of studies identifying gender-related differences in vaccine acceptance and uptake [22,34]. Subjects between 40 and 49 years old or those without children were less likely to be advocates. Vaccine advocates were more likely to have bachelor’s degrees and higher household incomes, a finding consistent with previous research [22]. In Hong Kong, vaccine acceptance was associated with pro-Beijing political leanings and good health status. Democratic voters were less likely to be vaccine advocates than pro-Beijing voters.

Age, education, political leaning, and health status all distinguished vaccine converts from resisters. Middle aged subjects (between 30 and 49) were less likely to convert, while well-educated subjects were more likely to convert. Vaccine converts were more likely to be pro-Beijing voters, and less likely to report poor health status. Public health authorities can use these findings to inform strategic communication. Based on the distinguishing characteristics of these different vaccination profiles, public health campaigns should be customized to target groups who are more likely to convert, including younger and older demographics, the well-educated, pro-Beijing voters, and those who report being in good health.

### 4.2. Psychological Determinants of Vaccine Advocates, Resisters, and Converts

Vaccine advocates were distinguished from vaccine resisters by being more trusting towards state government, distrusting of HK media, and demonstrating relatively low risk perception of the COVID-19 vaccine. Alternatively, those who converted to acceptance from resistance were more accepting of COVID-19 vaccine risks and reported increasing information consumption as well as increased risk perception related to the virus.

Responsibility for promoting public health-related messages lies with government, healthcare professionals, and media. Among vaccine resisters, however, high levels of distrust in state regulators and public health professionals render official messaging effectively useless. Alternative approaches for delivering vaccine-related messaging might utilize religious leaders or online opinion leaders [35,36,37]. Although trust in state officials increases vaccine uptake, trust in media leads to vaccine resistance. Intensive media coverage may discourage people from being vaccinated. The media should therefore report clear and unbiased information [1]. The opposite roles played by state and media messaging in vaccine acceptance suggests that institutions in Hong Kong did not work collaboratively to increase vaccination. Our results also demonstrated that resisters consume less vaccine-related information from public health professionals and pay less attention to COVID-related information on news media. This poses further challenges to the effective communication of accurate vaccine information to vaccine-resistant audiences. Alternative communication channels, such as local community and online forums [38], could increase the probability of reaching these individuals. 

Compared to vaccine resisters, vaccine converts demonstrated higher levels of confidence in COVID-19 vaccines. Many studies have emphasized the politicized nature of the COVID-19 pandemic and related vaccination campaigns [18]. Our research revealed a specific political differentiation between vaccine advocates and resisters, who varied in terms of their political leaning and their trust in authorities and institutions. For vaccine converts, however, pandemic- and vaccine-related perceptions were the determinant factors. Public health authorities should therefore place greater emphasis on educating the public about vaccines and mitigating their risk perception.

Our follow-up analysis (Supplementary Table S2) further suggested that confidence in vaccine was determined by trust in authorities. Confidence in vaccine at wave 2 was determined by vaccine confidence and trust in authorities at wave 1, as well as changes in trust and confidence between waves 1 and 2. This finding emphasized the total effect of individuals’ trust in public health systems. It suggested that addressing vaccine hesitancy requires more than increasing knowledge and building confidence in the vaccine. It is a multifactorial, evolving and context-dependent endeavor that requires a synchronous approach. Clear, consistent, multi-channel communication by health authorities about COVID-19 vaccine efficacy is crucial for building public confidence and improving vaccine uptake [2,39].

### 4.3. Limitations and Future Work

This study has several strengths, including its large sample size, a longitudinal data set that spans periods before and after the introduction of the vaccine, and a comprehensive profile of the psychology underpinning vaccine acceptance. However, these findings should be interpreted in light of several limitations. First, despite all efforts to make our sample inclusive and representative of Hong Kong’s adult population, we cannot rule out the possibility of potential biases due to quota sampling in the first wave and attrition in the second wave. Further, our analyses relied on self-reported vaccination status and psychological perceptions subject to recall and reporting bias. We also lack data on future vaccine uptakes. It is therefore difficult to know how changes observed during the study period might predict vaccination in the future. Despite these limitations, our time-lagged analysis still provides rigorous evidence for the roles played by psychological determinants of vaccination. In future studies, continued monitoring of vaccine uptake will help us to better understand changing vaccine intentions. For example, adding more longitudinal data in the future might allow us to see increases in groups that now remain relatively small, such as individuals who converted from intention to vaccinate to unvaccinated/vaccine resisters between waves 1 and 2. Finally, these results are based on a sample of adults in Hong Kong. Hong Kong citizens’ trust of state government, trust of media, attitudes towards vaccines, and perception of the pandemic may differ from other populations. Some determining factors are likely context dependent. Nevertheless, relationships found in our study can be generalized to other settings, e.g., the dominant role of trust for both vaccine confidence and vaccine uptake. Public health authorities in different nations could replicate our work to identify different psychological profiles in relation to the COVID-19 vaccine within their own populations, with the aim of tailoring campaign messages to these groups.

## 5. Conclusions

This study profiles the psychology underpinning different groups of test subjects and their differing attitudes toward the COVID-19 vaccine: vaccine advocates, converts, and resisters. We offer novel evidence regarding the importance of one’s psychological beliefs in determining acceptance of the COVID-19 vaccine, with an emphasis on individuals’ confidence in vaccine and trust in authorities. Previous studies investigating the psychological characteristics related to vaccine acceptance were based on cross-sectional surveys. Our longitudinal survey measured individuals’ psychological changes over time, offering insights into why individuals convert to vaccine acceptance or continue to insist on vaccine resistance. Based on our knowledge, this is the first study tracking individuals’ psychological changes during the pandemic as they pertain to vaccine intention. COVID-19 vaccination campaigns are ongoing throughout the world. Our results thus provide practical guidance for how to preserve current advocates and convert those who remain hesitant.

## Figures and Tables

**Table 1:** Sociodemographic characteristics of the Hong Kong sample (%).

	All (*n* = 1501)	Resister (*n* = 295)	Advocate (*n* = 458)	Convert (*n* = 621)
*Sex*				
Female	57.5	63.1	47.2	62.8
Male	42.5	36.9	52.8	37.2
*Age*				
20–29	13.3	10.5	10.3	16.9
30–39	28.2	30.5	24.7	29.6
40–49	24.9	29.2	22.7	24.6
50+	33.5	29.8	42.4	28.8
*Education*				
High school or less	28.9	36.3	28.8	24.5
Associate	14.4	15.3	14.6	13.4
Bachelor’s	42.2	34.9	42.8	45.9
Post-Graduate	14.5	13.6	13.8	16.3
*Household Income*				
<20 k	11.1	16.6	7.9	11.0
20–40 k	33.5	35.6	31.4	33.7
40–60 k	28.5	23.7	31.2	28.0
>60 k	26.8	24.1	29.5	27.4
*Residential Area*				
Hong Kong Island	17.3	17.6	17.5	17.2
Kowloon	31.7	34.2	30.6	31.2
New Territories	51.0	48.1	52.0	51.5
*Political Orientation*				
Pro-Democracy	27.6	35.3	18.6	29.0
Pro-Beijing	10.8	3.4	19.0	9.0
Centrist	11.6	6.1	15.3	11.3
Neutral	50.0	55.3	47.2	50.7
*Health Status*				
Good	45.7	37.3	52.2	46.2
Moderate	43.6	48.8	38.9	44.0
Poor	10.7	13.9	9.0	9.8

**Table 2:** Sociodemographic indicators associated with vaccine resisters, advocates, and converts in Hong Kong, 2020–2021.

	[Reference = Vaccine Resister]				[Reference = Vaccine Advocate]	
	Vaccine Advocate		Vaccine Convert		Vaccine Convert	
	AOR	95% CIs	AOR	95% CIs	AOR	95% CIs
*Sex (Female)*	**0.59**	**[0.43 0.81]**	0.98	[0.72 1.32]	**1.74**	**[1.35 2.26]**
*Age*						
18–29	-	-	-	-	-	-
30–39	0.65	[0.37 1.15]	**0.58**	**[0.35 0.95]**	0.92	[0.59 1.43]
40–49	**0.54**	**[0.30 0.97]**	**0.51**	**[0.30 0.85]**	0.97	[0.61 1.53]
50+	1.08	[0.61 1.94]	0.65	[0.39 1.10]	**0.63**	**[0.40 0.99]**
*No Children*	**0.57**	**[0.40 0.81]**	0.93	[0.66 1.31]	**1.64**	**[1.24 2.18]**
*Education*						
High school or less	-	-	-	-	-	-
Associate	1.27	[0.77 2.08]	1.33	[0.84 2.10]	1.11	[0.72 1.69]
Bachelor’s	**1.77**	**[1.17 2.68]**	**2.03**	**[1.38 2.96]**	1.20	[0.85 1.69]
Post-Graduate	1.30	[0.74 2.28]	**1.92**	**[1.15 3.20]**	1.57	[0.99 2.48]
*Household Income*						
<20 k	-	-	-	-	-	-
20–40 k	1.66	[0.98 2.81]	1.30	[0.83 2.05]	0.81	[0.50 1.30]
40–60 k	**2.35**	**[1.35 4.08]**	1.48	[0.91 2.41]	0.66	[0.41 1.09]
>60 k	**1.83**	**[1.03 3.26]**	1.27	[0.76 2.12]	0.75	[0.45 1.25]
*Residential Area*						
Hong Kong Island	-	-	-	-	-	-
Kowloon	0.96	[0.60 1.51]	0.97	[0.64 1.49]	1.00	[0.69 1.47]
New Territories	1.16	[0.75 1.79]	1.16	[0.78 1.73]	0.99	[0.69 1.41]
*Political Leaning*						
Neutral or None	-	-	-	-	-	-
Pro-Democrat	**0.46**	**[0.32 0.66]**	**0.67**	**[0.48 0.91]**	1.38	[1.00 1.92]
Pro-Beijing	**4.66**	**[2.32 9.35]**	**2.60**	**[1.28 5.27]**	**0.52**	**[0.35 0.78]**
*Health Status*						
Good	-	-	-	-	-	-
Moderate	**0.59**	**[0.43 0.83]**	0.74	[0.55 1.01]	1.22	[0.93 1.59]
Poor	**0.51**	**[0.31 0.86]**	**0.55**	**[0.34 0.88]**	1.00	[0.64 1.59]

*Note.* We conducted multinominal logistic regression (MLR) with sociodemographic factors as independent variables, with 95% confidence intervals (CIs) for the adjusted odds ratios (AOR). Statistically significant associations (*p* < 0.05) are highlighted in bold.

**Table 3:** Psychological indicators of vaccine advocates, resisters, and converts.

			Group Comparisons						
	Total		Vaccine Advocate ^a^		Vaccine Resister ^b^		Vaccine Convert ^c^		
	*n* = 1501		*n* = 458		*n* = 295		*n* = 621		
	Mean	SD	Mean	SD	Mean	SD	Mean	SD	ƞp^2^
**Wave 1**									
**[T1]** Politicization	4.92	1.37	**4.54 ^bc^**	1.31	**5.38 ^ac^**	1.34	**4.99 ^ab^**	1.34	0.051
**[T1]** Trust State	3.66	1.47	**4.50**	1.37	**2.87**	1.27	**3.42**	1.32	0.193
**[T1]** Trust Media	4.07	1.12	4.07	1.21	4.10	1.15	4.07	1.02	0.000
**[T1]** Trust Profs.	4.60	1.15	**4.67 ^b^**	1.12	**4.43 ^a^**	1.20	4.62	1.15	0.006
**[T1]** Information Acquisition	4.64	0.98	**4.92 ^bc^**	0.92	**4.40 ^a^**	1.04	**4.55 ^a^**	0.95	0.045
**[T1]** Media Attention	3.65	1.03	**3.87 ^bc^**	1.00	**3.53 ^a^**	1.10	**3.55 ^a^**	0.99	0.022
**[T1]** Perceived Risk	4.41	1.04	4.40	1.00	4.49	1.09	4.38	1.06	0.002
**[T1]** Worry	4.71	1.35	4.60	1.36	4.77	1.37	4.76	1.32	0.004
**[T1]** Vaccine Confidence									0.319
**Wave 2–Wave 1**									
**[T2-T1]** Politicization	−0.03	1.26	**0.17 ^b^**	1.32	**−0.27 ^ac^**	1.21	**−0.05 ^b^**	1.22	0.016
**[T2-T1]** Trust State	0.13	1.07	**0.08**	1.15	**0.06**	1.00	**0.21**	1.02	0.004
**[T2-T1]** Trust Media	−0.14	1.25	**0.02 ^bc^**	1.24	**−0.31 ^a^**	1.25	**−0.18 ^a^**	1.24	0.010
**[T2-T1]** Trust Profs.	−0.09	1.3	**0.19 ^bc^**	1.18	**−0.34 ^a^**	1.38	**−0.19 ^a^**	1.32	0.026
**[T2-T1]** Information Acquisition	−0.39	1.05	**−0.28 ^b^**	0.98	**−0.60 ^ac^**	1.17	**−0.37 ^b^**	1.04	0.012
**[T2-T1]** Media Attention	−0.18	1.06	−0.16	0.98	−0.27	1.13	−0.15	1.08	0.002
**[T2-T1]** Perceived Risk	−0.10	1.01	**0.04 ^b^**	1.00	**−0.37 ^ac^**	1.04	**−0.07 ^b^**	0.98	0.022
**[T2-T1]** Worry	−0.07	1.34	**0.10 ^bc^**	1.36	**−0.18 ^a^**	1.24	**−0.14 ^a^**	1.35	0.008
**[T2-T1]** Vaccine Confidence									0.111

*Note.* ANCOVAs were conducted with age, gender, income, and education as covariates. ƞp^2^, partial ƞ^2^. Turkey post-hoc analyses were conducted to compare the three groups. ^abc^ = mean difference between denoted categories is significant at the 0.05 level. Statistically significant comparisons are highlighted in bold. Trust profs. = trust public health professionals.

## Data Availability

Data are contained within the article or Supplementary Material.

## Supplementary Materials

The following supporting information can be downloaded at: https://www.mdpi.com/article/10.3390/vaccines10101744/s1, Figure S1: Geolocation distribution of our sample in Hong Kong; Figure S2: Theoretical framework; Table S1: Comparison of sample demographics with Hong Kong census figures; Table S2: Zero-order and partial associations for vaccine acceptance with psychosocial variables; Table S3: Multiple linear regression and binominal logistic regression explaining vaccine risk acceptance and vaccination status at wave 2; Table S4: The Measurement Scale.
