# Supplementary material for: Increasing Vaccination: Psychological Characteristics of COVID-19 Vaccine Advocates, Converts, and Resisters in Hong Kong
Source: Vaccines (Basel). 2022 Oct 19;10(10):1744. doi: 10.3390/vaccines10101744 (PMC9611256; doi:10.3390/vaccines10101744)
Supplement: Supplementary file 1 [file vaccines-10-01744-s001.zip › vaccines-1970373-supplementary.pdf]

Supplementary Information

Increasing vaccination: Psychological characteristics of COVID-19 vaccine advocates, converts, and resisters in Hong Kong

Supplementary Methods

Sample Characteristics and Covariates

Sociodemographic characteristics for this study included gender, age, education, income, political leanings, residence, and health status. Women accounted for 57.5% of the sample. Age was measured on an 8-point scale ranging from 1 = *20 - 24 years old* to 8 = *over 55 years old* (M = 5.14, SD = 2.17). These eight age ranges were then coded into four categories. In terms of residential areas, over half (51.03%) of respondents lived in New Territories, 31.71% were from Kowloon, and 17.3% were from Hong Kong Island (Supplementary Figure 1). Respondents were asked to report their highest education level on a 7-point scale with 1 = *junior high school or below* and 7 = *doctorate* (M = 4.09, SD = 1.55). Monthly household income was measured using a 7-point scale with 1 = *HK$10,000 or below* and 7 = *above HK$60,000* ($M$ = 4.81, SD = 1.76). Politically, 49.17% were apolitical, 19.19% were democrats, and 10.86% leaned pro-Beijing. In addition, respondents were asked to self-report their general perceptions of their own health from 1 = *very poor* to 7 = *very good* (M = 4.55, SD = 1.06). This variable was recoded to three conditions: good, moderate, and poor health status. Supplementary Table 1 shows the gender, age, and residence distributions of our sample compared to 2016 Hong Kong census data.[1]

Compared to the census data, our two-wave sample leaned more female (χ^2 = 14.08, $p < .05$) and middle-aged (χ^2 = 235.33, $p < .05$), with higher income (χ^2 = 694.96, $p < .05$) and higher reported education levels (χ^2 = 1384.26, $p < .05$).

Table S1. Comparison of sample demographics with Hong Kong census figures.

	Hong Kong 2016 Population By-census	Wave 1 Study (n=3,190)	Wave 2 Study (n=1,501)
Gender			
Male	47%	43.4%	42.5%
Female	53%	56.6%	57.5%
Age			
20-24	7.46%	9.2%	3.70%
25-29	7.91%	11.5%	9.70%
30-34	8.50%	13.0%	14.10%
35-39	8.38%	12.9%	14.20%

[1] https://www.censtatd.gov.hk/hkstat/sub/sp459.jsp

40-44	8.92%	11.8%	14.90%
45-49	9.22%	8.9%	10.10%
50-54	10.70%	12.0%	10.70%
55+	38.90%	20.7%	22.90%
Residence			
Hong Kong Island	17%	17.5%	17.3%
Kowloon	31%	32.1%	31.7%
New Territories	53%	50.4%	51%

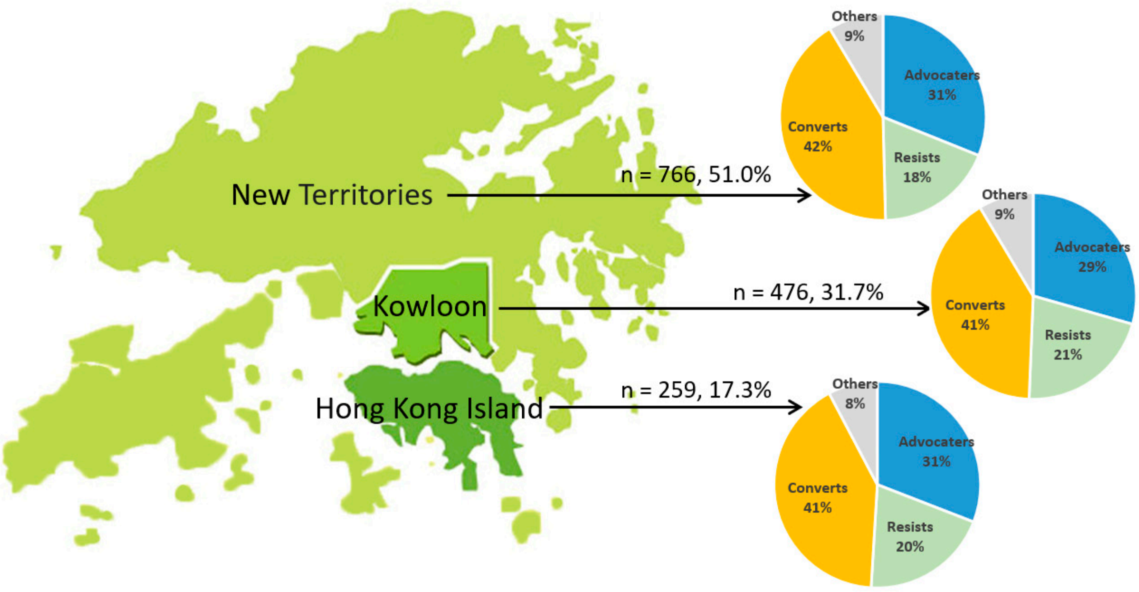

Figure S1. Geolocation distribution of our sample in Hong Kong.

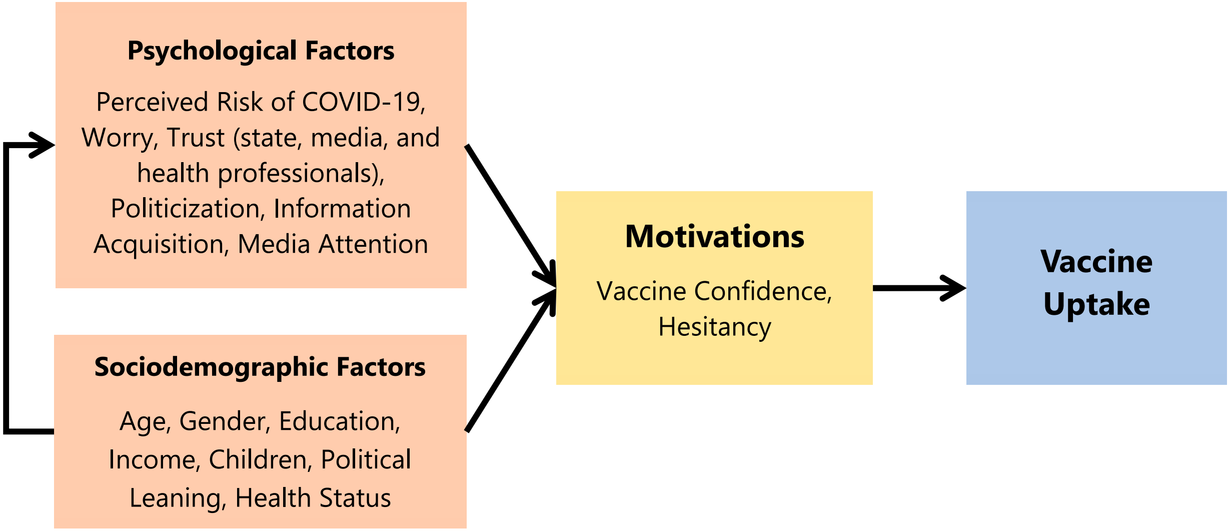

Figure S2. Theoretical framework

Supplementary Results

Table S2. Zero-order and partial associations for vaccine acceptance with psychosocial variables.

		**[T2] Vaccine Risk Acceptance**		**[T2] Vaccination**	
	Mean (*SD*)	*r*	Spearman *r* partial	Point-biserial *r*	AOR
**Wave 1**					
[T1] Politicization	4.93 (1.37)	**-0.25**	**-0.20**	**-0.14**	**0.77**
[T1] Trust in State	3.67 (1.47)	**0.40**	**0.35**	**0.21**	**1.48**
[T1] Trust in Media	4.10 (1.13)	-0.03	0.01	-0.05	0.91
[T1] Trust in Professionals	4.61 (1.16)	0.09	0.07	0.05	1.08
[T1] Perceived Risk	4.44 (1.04)	-0.04	-0.04	-0.09	0.83
[T1] Worry	4.74 (1.35)	**-0.11**	**-0.07**	-0.06	0.90
[T1] Information Acquisition	4.65 (0.97)	**0.20**	**0.21**	0.09	1.20
[T1] Media Attention	3.67 (1.04)	0.07	0.08	0.00	1.00
[T1] Vaccine Confidence	3.99 (1.29)	**0.37**	**0.33**	**0.17**	**1.33**
[T1] Intention to Vaccination	3.82 (1.41)	**0.40**	**0.34**	**0.21**	**1.40**
**Wave 2 – Wave 1**					
[T2-T1] Politicization	-0.04 (1.26)	**0.13**	**0.13**	-0.09	1.17
[T2-T1] Trust in State	0.15 (1.07)	**0.11**	**0.11**	0.02	1.05
[T2-T1] Trust in Media	-0.15 (1.25)	**0.13**	**0.08**	0.05	1.09
[T2-T1] Trust in Professionals	-0.09 (1.29)	**0.21**	**0.18**	0.07	1.13
[T2-T1] Perceived Risk	-0.10 (1.00)	**0.15**	**0.10**	**0.11**	**1.29**
[T2-T1] Worry	-0.08 (1.33)	0.09	0.07	0.03	1.06
[T2-T1] Information Acquisition	-0.39 (1.05)	0.09	0.06	0.07	1.14
[T2-T1] Media Attention	-0.19 (1.06)	0.06	0.04	0.04	1.08
[T2-T1] Vaccine Confidence	0.23 (1.51)			**0.29**	**1.60**

Note. [T1] = variables measured at wave 1, [T2] = variables measured at wave 2, [T2-T1] = changes between wave 2 and wave 1. "Trust in Professionals" refers to trust in healthcare professionals. To avoid capitalizing on chance, p-values are not presented. Given that this study was designed to detect small to medium effects over a one-year period, correlations > 0.1 are assumed to be non-trivial. Partial correlations and adjusted odds ratios are presented, with controls for wave 1 age, gender, education, and income.

Table S3. Multiple linear regression and binominal logistic regression explaining vaccine risk acceptance and vaccination status at wave 2.

	[T2] Vaccine Risk Acceptance		[T2] Vaccination	
	(s.e.)	*p*-value	AOR	95% CIs
Intercept	**1.98 (0.32)**	0.000	0.07	[0.01, 0.31]
[T1] Politicization	0.02 (0.03)	0.37	1.03	[0.87 1.21]
[T1] Trust in state	**0.39 (0.04)**	0.000	0.97	[0.80 1.18]
[T1] Trust in media	**-0.15 (0.03)**	0.000	**0.81**	**[0.67 0.99]**
[T1] Trust in professionals	**0.10 (0.03)**	0.001	0.92	[0.76 1.12]
[T1] Perceived risk	-0.04 (0.04)	0.15	1.02	[0.83 1.26]
[T1] Worry	0.03 (0.03)	0.34	0.94	[0.78 1.14]
[T1] Information Acquisition	0.01 (0.03)	0.88	1.13	[0.90 1.41]
[T1] Media Attention	0.01 (0.03)	0.86	0.97	[0.80 1.17]

[T2-T1] Politicization	0.04 (0.03)	0.13	1.14	[0.98 1.32]
[T2-T1] Trust in state	**0.25 (0.03)**	0.000	0.86	[0.71 1.06]
[T2-T1] Trust in media	**-0.07 (0.03)**	0.01	0.89	[0.76 1.05]
[T2-T1] Trust in Professionals	**0.10 (0.03)**	0.002	0.84	[0.71 1.00]
[T2-T1] Perceived risk	0.04 (0.02)	0.15	1.19	[0.96 1.33]
[T2-T1] Worry	0.01 (0.03)	0.78	0.90	
[T2-T1] Information Acquisition	-0.01 (0.03)	0.61	1.16	[0.96 1.39]
[T2-T1] Media Attention	0.03 (0.03)	0.35	1.00	[0.84 1.19]
[T1] Risk Acceptance	**0.23 (0.02)**	0.000	**2.75**	**[2.16 3.52]**
[T2-T1] Vaccine Confidence			**3.16**	**[2.71 3.70]**
[T1] Vaccination Intention			1.13	[0.95 1.36]
	$R^2 = 37.1\%$, $p < 0.001$		*Pseudo* $R^2 = 28.6\%$, $p < 0.001$	

Supplementary Table S4. The Measurement Scale.

**Measurements**
**Vaccination Intention (Wave 1 Only)**<br>If the disease control and prevention authorities developed a vaccine to deal with COVID-19, how likely would you be to take the following actions? 如果現在疾病防控部門研發出新型冠狀病毒肺炎的疫苗，您有多大可能採取以下行動?<br><br>(1) I would get the vaccine sometime soon. 我會在最近幾天接種疫苗<br>(2) If I were faced with the decision of whether to get the vaccine today, I would choose to get it. 如果今天必須作出決定是否接種疫苗，我會進行接種<br>(3) I would get the vaccine in the future. 我會在未來接種疫苗
**Vaccination Status (Wave 2 Only)**<br>What is your COVID-19 vaccination status? 目前為止您的新冠疫苗接種情況是?<br><br>(1) I don't plan to receive the vaccine. 沒計畫接種疫苗<br>(2) I have scheduled to be vaccinated but not yet vaccinated. 已經預約接種疫苗但還未接種<br>(3) I have had my first shot of the vaccine. 已接種第一針疫苗<br>(4) I have had my first shot of the vaccine (only one-shot required, e.g., J&J vaccine). 已接種第一針疫苗（只需接種一針，如強生疫苗）<br>(5) I've had my first dose of the vaccine and don't plan to get the second dose. 已接種第一針疫苗，且不打算接種第二針<br>(6) I have had my second shot of the vaccine. 已接種第二針疫苗
**Vaccine Confidence**<br>To what extent do you agree with the following statements regarding the COVID-19 vaccines? 對於接種新冠疫苗，您有多大程度同意以下各項描述?<br><br>(1) I can accept the potential risks of the vaccine. 我可以接受接種疫苗存在的風險<br>(2) I am highly willing to accept the potential risks of the vaccine. 我對接種疫苗存在的風險接受程度<br>(3) After careful deliberation, I think the vaccine does more good than harml. 經過仔細考慮，我認為接種疫苗利大於弊
**Perceived Risk**<br>With respect to the risk that arises from COVID-19, how much do you agree with the following statements? 對於 2019 冠狀病毒帶來的風險，您有多大程度同意以下各項描述?

(1) The coronavirus is almost ubiquitous, and the pathogenicity is high. 該病毒幾乎無處不在，很容易染病
(2) There is a high probability of getting infected. 感染機會很大
(3) The mortality rate of COVID-19 is high. 染病的死亡率很高
(4) I have no confidence I can avoid the negative impacts of COVID-19. 我沒有把握避開該風險帶來的負面影響

Worry
With respect to the risk that arises from COVID-19, how much do you agree with the following statements? 對於 2019 冠狀病毒帶來的風險，您有多大程度同意以下各項描述?

(1) I feel fearful. 我感到害怕
(2) I feel sad. 我感到悲傷
(3) I feel helpless. 我感到無助
(4) I feel worried. 我感到擔心

Trust in State
How much do you trust the following institutions/people?
您有多大程度信任以下機構或人員?

(1) State government 特區政府
(2) The Food and Health Bureau 食物及衛生局
(3) The Center for Health Protection 衛生防護中心

Trust in Media
How much do you trust the following institutions/people?
您有多大程度信任以下機構或人員?

(1) Hong Kong media 香港媒體

Trust in Public Health Professionals
How much do you trust the following institutions/people?
您有多大程度信任以下機構或人員?

(1) Public health professionals 相關醫療專家

Information Acquisition
How long do you use the following media channels on average per day? 您平均每天使用多長時間在以下媒體渠道?

(1) Television 電視
(2) Newspapers 報紙
(3) Websites (e.g., Yahoo) 網站 (如雅虎等)
(4) Social media (e.g., WhatsApp, Facebook) 社交媒體 (如 WhatsApp, Facebook 等)
(5) Short video platforms (e.g., Tik Tok) 短視頻平台 (如 Tik Tok 等)

Media Attention
How often do you pay attention to information about COVID-19 when using the following media channels? 請問您通過以下方法關注 2019 冠狀病毒肺炎資訊的程度是?

(1) Television 電視
(2) Newspapers 報紙
(3) Websites (e.g., Yahoo) 網站 (如雅虎等)
(4) Social media (e.g., WhatsApp, Facebook) 社交媒體 (如 WhatsApp, Facebook 等)
(5) Short video platforms (e.g., Tik Tok) 短視頻平台 (如 Tik Tok 等)

Issue Politicization

With respect to COVID-19, how much do you agree with the following statements? 提及 2019 冠狀病毒肺炎事件，您有多大程度同意以下各項描述?

(1) It is too mixed up in politics. 有關事件的發展與政治因素密不可分

(2) Due to political interests, policy decisions have favored some groups. 相關決策因政治利益偏袒了某些團體

(3) It is politically motivated. 有關事件的處理存在政治動機

(4) Political considerations affect the nature of the information that the public receives about this issue. 政治因素影響了公眾所接收到的資訊內容
